# Bacterial Adhesion on Femtosecond Laser-Modified Polyethylene

**DOI:** 10.3390/ma12193107

**Published:** 2019-09-24

**Authors:** Karin Schwibbert, Friederike Menzel, Nadja Epperlein, Jörn Bonse, Jörg Krüger

**Affiliations:** Bundesanstalt für Materialforschung und -prüfung (BAM), Unter den Eichen 87, D-12205 Berlin, Germany; friederike_menzel@web.de (F.M.); nadja.epperlein@web.de (N.E.); joern.bonse@bam.de (J.B.); joerg.krueger@bam.de (J.K.)

**Keywords:** bacterial adhesion, biofilm formation, laser-modified surface, polyethylene, laser-induced nanostructures

## Abstract

In this study, femtosecond laser-induced sub-micrometer structures are generated to modify polyethylene (PE) surface topographies. These surfaces were subjected to bacterial colonization studies with *Escherichia coli* and *Staphylococcus aureus* as test strains. The results reveal that the nanostructures do not influence *S. aureus* coverage, while the adhesion of *E. coli* is reduced.

## 1. Introduction

Bacteria mostly live attached to surfaces rather than suspended in aqueous solutions [1,2]. Primary bacterial adhesion is followed by cell proliferation and production of extracellular polymeric substances (EPS), which are usually major constituents of a mature biofilm. This matrix encases the cells, thereby forming water channels that help to distribute nutrients and offering protection against organic solvents and antibiotics [3,4,5]. In most cases, biofilm formation is detrimental to industrial processes, it may cause corrosion, clogging and reservoir souring e.g., in food production or water distribution systems [6,7,8]. These bacteria are very often hazardous to human health [4,9,10]. All these negative aspects of biofilms seem to overweigh the positive aspects of the robust nature of biofilms, despite they do exist in settings where whole cells find useful applications in, e.g., soil bioremediation or waste water treatment [11,12].

Hence, the control of biofilm formation on surfaces is of major concern and numerous efforts have been made to render surfaces antimicrobial [13,14]. Three different mechanisms of antimicrobial action of surfaces can be distinguished: (I) surfaces that release antimicrobial agents, (II) contact-killing surfaces that kill bacteria upon adhesion without release of antimicrobials, and (III) surfaces that prevent bacterial adhesion. Chemical-based approaches that depend on the release of antibiotics from coatings or substrates are widespread. However, the gradual loss of antibiotics implies that their concentrations, though highly toxic at first, will decrease to a non-lethal level over time, which may finally promote the development of antibiotic resistance [15]. Therefore, passive surfaces acting through topographic rather than chemical means are considered advantageous for long-term antimicrobial or antiadhesive effects. Micro- and nanoscale topography of a surface is known to influence bacterial adhesion behavior and, in addition, may exert a mechano-bactericidal effect, which does not prevent adhesion, but kills bacteria as soon as they attach to the surface [9,16,17,18,19,20,21,22,23,24,25]. To introduce micro- and nanoscale structures onto a surface, laser-based techniques are increasingly gaining importance through Direct Laser Interference Patterning (DLIP) [26,27,28] and the generation of laser-induced periodic surface structures (LIPSS) [29,30,31].

LIPSS, often termed ripples, are a universal phenomenon manifesting as a quasi-periodic topographic surface relief. They can be generated on almost any material upon irradiation with linearly polarized laser radiation. Usually, they are formed in a specific range of laser processing parameters featuring energy densities (fluences in J/cm^2^) close to the material related melting/ablation threshold. LIPSS were generated on a variety of polymers using pulse durations in the picosecond and femtosecond range [32,33,34].

Polyethylene is a material with an extreme wide range of applications. The global annual production is more than 100 million tons which makes it one of the world’s most significant kind of plastic [35]. Application fields cover public water supply (pipes for drinking and processing water), typical household plastics like bottles or packaging plastics, and medical devices like syringes, tubings, urine or blood bags, and also artificial hip joints [36,37], only to mention a few. Due to the inert and unreactive character of polyethylene, surface modification of PE with the aim to reduce bacterial adhesion is, therefore, especially of medical interest and a worthy goal.

So far, efforts to develop polyethylene with antimicrobial properties mainly concentrated on melt compounding with biocides following the above-mentioned strategy I [38,39,40]. In contrast, less work was performed using strategies II and III, i.e., reducing the bacterial growth/adhesion through surface topographical changes. Here, we report the femtosecond laser-based manufacturing of large-area sub-micrometer surface structures on polyethylene. The topographical feature sizes were chosen since they match with the dimensions of typical bacterial cells, allowing to test specifically strategy III. The non-irradiated and laser-irradiated samples were subjected to microbial adhesion tests with *Escherichia coli* and *Staphylococcus aureus* as medically relevant test strains. The question is addressed if the generated nanostructures on polyethylene noticeably influence the adhesion behavior of bacteria with different cell shapes.

## 2. Materials and Methods

High-density polyethylene specimens were prepared from commercial polymer granules by compression molding at the Bundesanstalt für Materialforschung und -prüfung (BAM). A detailed description of the production process and material properties of the PE-HD 1 samples can be found in [41].

### 2.1. Laser Processing

A commercial Ti:sapphire femtosecond (fs) laser system (Compact Pro, Femtolasers, Vienna, Austria) emitting linearly polarized laser pulses with a pulse duration of 30 fs, a center wavelength of 790 nm and a pulse repetition rate of 1 kHz was utilized for surface processing. The Gaussian laser beam was focused with a spherical dielectric mirror of 500 mm focal length, resulting in a focused beam diameter (at 1/e^2^) of about 90 µm. With a pulse energy of 40 µJ, a peak fluence of 1.2 J/cm^2^ was realized. The samples were placed on computer-controlled *x*-*y*-*z* linear translation stages under normal incidence. During the laser processing of an area of 5 × 5 mm^2^, the sample was moved at a constant scan velocity of 2.4 mm/s in a line-wise meandering way. The separation between individual lines was 2.4 µm. The number of effective pulses per beam spot diameter was ∼40 in the direction of laser scanning. For the proper selection of the samples for the bacterial colonization tests, sample surfaces were characterized using optical microscopy (OM, Nikon Eclipse L200, Tokyo, Japan).

### 2.2. Contact Angle Measurement

The wetting behavior of the PE surfaces was determined by contact angle measurements (sessile drop technique) using the Drop Shape Analyser DSA30 (Krüss, Hamburg, Germany) with the corresponding software (Krüss Advance). Deionized water droplets with 1 µL volume were displaced on the laser-irradiated and non-irradiated areas automatized allowing the droplet to settle. At three representative sample sites per surface modification a droplet was set, and at least four measurements were carried out automatically on the same droplet resulting in an averaged contact angle (CA).

### 2.3. Microorganisms, Culture Conditions, and Bacterial Adhesion Assays

*Escherichia coli* TG1 (DSM6056) and *Staphylococcus aureus* (BAM480) were chosen for adhesion experiments as representatives of rod-like gram negative and spherical gram positive bacteria. Two samples per surface conditioning (laser-irradiated and non-irradiated) were tested per organism. For *E. coli*, 20 mL LB (Luria-Bertani) medium ([42], p. 1087) was inoculated with single colonies and incubated overnight at 37 °C on an orbital shaker at 120 rpm. This preculture was diluted 1:10 in prewarmed LB and incubated again at 37 °C with shaking at 120 rpm until the cells reached the exponential growth phase (OD600 0.4–0.6). Then cells were collected by centrifugation (2 min, 5000 g), washed with M9 medium ([42], p. 1135) which was supplemented with 1 mM thiamine and proline 20 mg/L (M9 + Thi/Pro). Cell suspension was then diluted to 10^7^ cells/mL in M9 + Thi/Pro.

For *S. aureus*, the same procedure was carried out, except that tryptic soy broth (TSB) was used throughout the whole procedure and, therefore, the washing step was omitted. Furthermore, incubations were carried out at 30 °C.

The samples were washed in detergent solution, disinfected for 5 min in 70% ethanol, air-dried and transferred to sterile 6-well plates. 7 mL of the bacterial suspensions (10^7^ cells/mL) were added. After an initial sedimentation phase of 1 h at 37 °C or 30 °C (*E. coli* or *S. aureus*) the plates were further incubated with shaking at 60 rpm. After 3 h and 21 h samples were removed and subjected to the following washing procedure to remove lightly attached cells: Samples were immersed in 8 mL sterile phosphate buffered saline (PBS) solution (8.0 g NaCl, 0.2 g KCl, 1.44 g Na_2_HPO_4_, 0.24 g KH_2_PO_4_) in a sterile 6-well plate. After one minute, 4 mL PBS were removed and replaced by the same volume of fresh PBS under gentle shaking. These steps were repeated twice.

After fixation with 4% glutaraldehyde in PBS at room temperature, the samples were again subjected to the washing procedure, then dehydrated using gradient ethanol solutions of 30, 50, 70, 80, 90% (v/v), and ethanol absolute for 20 min, followed by critical point drying with carbon dioxide (EM CPD300, Leica, Wetzlar, Germany).

### 2.4. Environmental Scanning Electron Microscopy and Data Analysis

Dried samples were coated with a 15 to 30 nm conducting layer of gold (EM ACE600, Leica) and investigated with an environmental scanning electron microscope (FEI XL 30, Hillsboro, OR, USA) equipped with a secondary electron detector. Surface areas covered with adherend bacteria were calculated using the Fiji software (2017 May 30) [43]. A minimum of three random fields with a total surface area of at least 15,000 µm^2^ were analyzed per sample.

## 3. Results

### 3.1. Laser Surface Processing of Polyethylene

**Figure 1:**
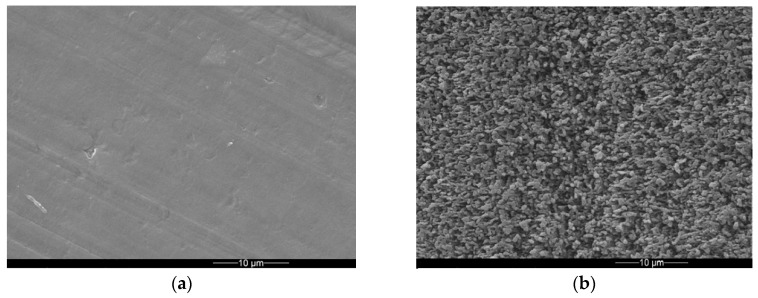
Environmental scanning electron micrograph of a pristine PE surface (**a**) and the corresponding femtosecond laser-processed PE surface (**b**).

An environmental scanning electron micrograph (ESEM) of the pristine PE surface is depicted in Figure 1a, indicating a smooth surface with a shallow preferential structure arising from sample preparation by compression molding. After femtosecond laser processing, the surface appears roughened exhibiting irregularly arranged sub-micrometer topographical features (Figure 1b).

The wetting behavior with water of both characteristic surfaces shown in Figure 1 was characterized by contact angle measurements. The data are compiled in Table 1. Obviously, the PE surface turns from a hydrophilic to a hydrophobic state upon femtosecond laser processing. This may be affected by both, topographical and chemical alterations of the PE surface.

### 3.2. Bacterial Adhesion and Biofilm Formation on PE Surfaces

**Figure 2:**
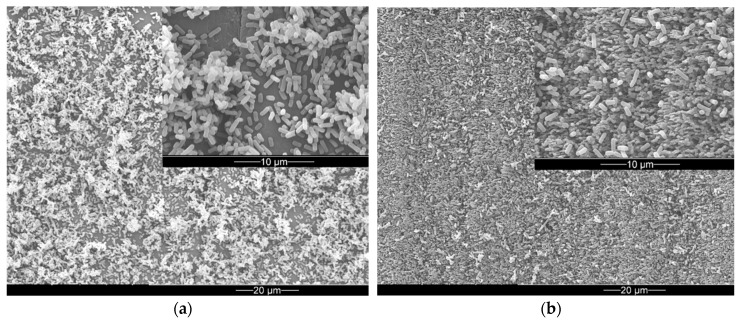
Environmental scanning electron micrograph (ESEM) of *E. coli* after 21 h co-cultivation with PE sample (**a**) on a pristine surface area (**b**) on a laser-processed surface area. The insets present high-resolution micrographs.

*E. coli* and *S. aureus* were used as test strains. Bacteria were co-incubated with PE samples for short time (3 h) or overnight (21 h). As depicted in Figure 2a, after 21 h of cultivation the rod-shaped *E. coli* cells displayed extensive and dense adhesion in multi-layers after overnight incubation on pristine PE surface. In contrast, on laser-processed PE surface, adhesion of *E. coli* was significantly lower. No multi-layers have formed, only single separated cells are dispersed on the surface (Figure 2b).

**Figure 3:**
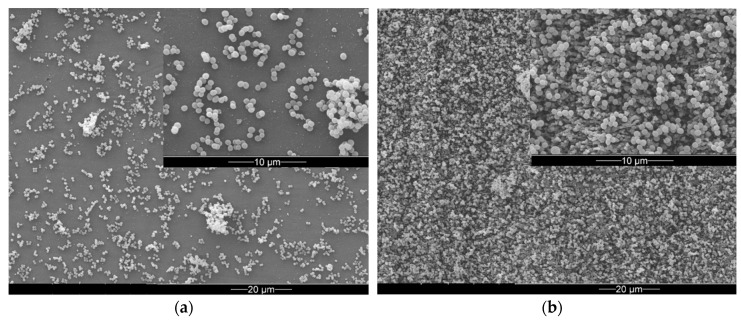
ESEM of *S. aureus* after 21 h co-cultivation with PE sample (**a**) on a pristine surface area (**b**) on a laser-processed surface area. The insets present high-resolution micrographs.

Adhesion behavior of the spherical-shaped *S. aureus* cells was completely different. Here, after a cultivation period of 21 h a biofilm with grape-like cell clusters developed over the whole sample, regardless of the presence of laser-induced nanostructures (compare Figure 3a,b). 

**Figure 4:**
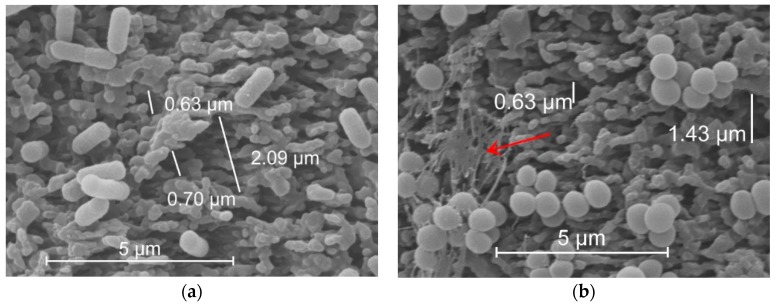
ESEM of *E. coli* (**a**) and *S. aureus* (**b**) after 21 h co-cultivation with PE sample on a laser-processed surface area. The arrow in (**b**) indicates EPS matrix. Distance measurements were performed with Nikon Imaging Software NIS-Elements 4.20.

One reason for the observed differences in colonization pattern might be the dimensions of the laser-induced nanostructures relative to the cell shapes. As depicted in Figure 4, valley sizes of the processed surface ranged from 0.6 to 2 µm in diameter. These dimensions fit perfectly to host spherical *S. aureus* cells, which are 0.5–1 µm in diameter. Compared to *S. aureus*, surface area accessible for adhesion is reduced for *E. coli*, because the rod-shaped *E. coli* cells are too long (>1 µm) to adhere to small valleys. A large proportion of *E. coli* cells lies on top of protruding structures, therefore, the contact area is too small for strong and extensive adhesion on the laser-processed surface. In addition, *S. aureus* is a typical biofilm former which produces an EPS matrix very easily (Figure 4b). This matrix promotes collective bacterial adhesion and also masks partially the laser-generated micro-topography on the sample surface.

**Figure 5:**
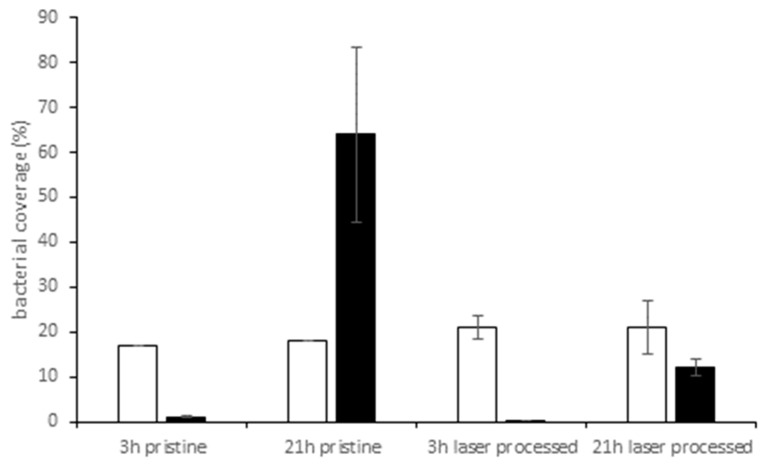
Bacterial coverage (%) on pristine or laser processed PE samples after co-cultivation for 3 h or 21 h. □ *S. aureus*, ■ *E. coli* (weighted mean values and standard deviations, repetitions: obtained from five images for *S. aureus*, obtained from three images for *E. coli*). Note that for the pristine surfaces, the standard deviation is negligible since the bacteria were evenly distributed on the surface.

The influence of cultivation time on the colonization pattern of the two test strains is shown in Figure 5. After 3 h, bacterial coverage was 1% or below for *E. coli* on both surfaces, pristine or laser-processed. After overnight cultivation, *E. coli* covered 64% of pristine and 12% of the laser-processed surface. Distribution of *E. coli* cells on pristine surface was remarkable uneven after 21 h, indicated by the large standard deviation obtained from these data. For *S. aureus*, we noted a considerable and almost constant coverage of 17–20% on pristine or laser-processed samples after short time or overnight incubation.

## 4. Discussion

In the present work, we analyzed the possibility of using femtosecond laser surface structuring as a method to control the colonization of polyethylene surfaces by bacteria. The femtosecond laser treatment, reliably and contactless adds a nanoscale roughness to the PE surface, increasing its hydrophobicity. This can be seen by a change of the contact angle of water from ~65° to ~120° after the laser processing. It is known that it is not only the topography, but also differences in surface hydrophobicity that might influence bacterial colonization [44,45,46,47]. So far, results of bacterial adhesion assays on surfaces with different wettability are contradictory, mainly because of the different test procedures employed which could significantly affect the results [44]. In our study, both bacterial strains adhered extensively to the pristine, hydrophilic areas, whereas the laser-structured, hydrophobic areas seemed to repel *E. coli* but not *S. aureus*. Here, this behavior cannot be attributed to differences in wettability alone but also points out the influence of surface morphology. 

It is also reported that the capability of superhydrophobic surfaces to reduce bacterial adhesion will decrease under submerged conditions over time after the trapped air is completely lost [48,49] and at this point, surface topography influences bacterial adhesion much stronger than surface wettability [50]. During our test procedure, the samples were submerged in bacterial suspension for up to 21 h and, therefore, we assume, that in our case, the influence of surface morphology on adhesion characteristics of the test strains dominates over wettability. 

It is well known that microscale topography like grooves, ripples, and spikes introduced by various technologies influence bacterial colonization and biofilm formation by providing different opportunities for bacterial adhesion [17]. Our results show that the surface topography introduced on polyethylene by femtosecond laser treatment prevents adhesion of *E. coli* but this is not the case for *S. aureus*. It is assumed that cells try to maximize contact area with the surfaces presumably to achieve a more stable attachment [51]. Although the total surface area increased due to the laser treatment in our experiments, this area is not completely available for bacterial adhesion. The actual possible adhesion area strongly depends on the shape of bacteria under investigation that might present a steric hinderance. Fadeeva et al. reported different adhesion characteristics of *S. aureus* and *Pseudomonas aeruginosa* cells on a femtosecond laser-processed titanium surface mimicking the lotus leaf and attributed this to differences in the cell shapes [52]. The same arguments were brought in by Epperlein et al. who proposed that differences in cell shape is mainly responsible for differences in adhesion behavior observed for *E. coli* and *S. aureus* on femtosecond laser-structured steel [53]. 

Bacteria tend to choose valleys instead of protruding features for adhesion, if possible [54]. In our study, spherical *S. aureus* (typically 0.5–1 µm in diameter) is obviously small enough to find niches on the nanostructured area where the characteristic spacing between protrusions of approximately 0.6–2 µm fits the cell’s shape and, therefore, promotes adhesion. The rod-like *E. coli* cells (typically 1–3 µm long and 0.5–1 µm wide) on the contrary do not fit to that length scale (see Figure 4). These findings are in line with the results of Gu et al., who documented a better inhibition of *E. coli* adhesion on surfaces with smaller inter-pattern spacing [55] and Epstein et al., who reported on a mechano-selective attachment of bacteria on compliant high-aspect-ratio nanostructures [56]. In sum, microscale surface topographies may inhibit or promote bacterial attachment depending on size, shape and density of the features.

The work of Friedlander et al. demonstrated that adhesion to topographic surfaces with trenches smaller than the cell body was significantly reduced during the first 2 h compared with flat controls but this behavior reversed with longer incubation times [57]. Our results, however, demonstrated lower coverage values of *E. coli* on the nanostructured area compared to a flat surface after short and longtime incubation (3 h and 21 h, see Figure 5) Adhesion of *S. aureus* was not affected to any extent by laser-induced nanostructures and we assume that the influence of EPS dominates the adhesion behavior.

It is known that bacterial adhesion is a very complex process and dictated by a number of variables, including the species of bacteria, chemical surface composition, and environmental factors. Differences in adhesion behavior in our study in comparison to the results reported by Friedlander et al. [57] might therefore be attributed to differences in the structured surfaces (regular grids with 2.7 µm in height, 3 mm in diameter, 440 nm inter-pattern space versus irregular), in material (PDMS polymer versus PE), culture conditions (use of different growth media, static versus gentle rotation at 60 rpm) or test strains. 

Overall, the femtosecond laser processing technique proved to be powerful for influencing bacterial colonization on polyethylene. Specifically, it was demonstrated, that adhesion of *E. coli* was significantly impeded on the laser-processed area featuring nanoscale roughness compared to the pristine surface. Due to the huge number of variables that influence the interplay between bacterial cells and surfaces, it seems almost impossible to fabricate a surface with a topography, which prevents adhesion of all bacteria in all environments. Even if 99% bacteria are prevented from attachment to a surface, the remaining 1% will adhere, propagate, and eventually die, leaving a dead biomass as breeding ground for the next generation. Antifouling designs should, therefore, focus on a combination of the specific demands the application environment poses on the material surface and at easy to clean surfaces including a cleaning procedure. 

## Figures and Tables

**Table 1:** Averaged contact angles of water on pristine and laser-modified PE surfaces. In both cases, the standard deviation of the contact angle was below 2° (number of measurements ≥ 12).

Surface	Contact Angle
Pristine polyethylene	64.5°
Femtosecond laser-processed polyethylene	120.5°

## References

[1] Zobell C.E. (1943). The Effect of Solid Surfaces upon Bacterial Activity. J. Bacteriol..

[2] Costerton J. (1999). Introduction to biofilm. Int. J. Antimicrob. Agents.

[3] Donlan R.M. (2002). Biofilms: Microbial Life on Surfaces. Emerg. Infect. Dis..

[4] Dufour D., Leung V., Levesque C.M. (2012). Bacterial biofilm: Structure, function, and antimicrobial resistance. Endod. Top..

[5] Luppens S.B.I., Reij M.W., Van Der Heijden R.W.L., Rombouts F.M., Abee T. (2002). Development of a Standard Test to Assess the Resistance of Staphylococcus aureus Biofilm Cells to Disinfectants. Appl. Environ. Microbiol..

[6] Xu D., Jia R., Li Y., Gu T. (2017). Advances in the treatment of problematic industrial biofilms. World J. Microbiol. Biotechnol..

[7] Galié S., García-Gutiérrez C., Miguélez E.M., Villar C.J., Lombó F. (2018). Biofilms in the Food Industry: Health Aspects and Control Methods. Front. Microbiol..

[8] Chan S., Pullerits K., Keucken A., Persson K.M., Paul C.J., Rådström P. (2019). Bacterial release from pipe biofilm in a full-scale drinking water distribution system. NPJ Biofilms Microbiomes.

[9] Bixler G.D., Bhushan B. (2012). Biofouling: Lessons from nature. Philos. Trans. R. Soc. A Math. Phys. Eng. Sci..

[10] Di Pippo F., Di Gregorio L., Congestri R., Tandoi V., Rossetti S. (2018). Biofilm growth and control in cooling water industrial systems. FEMS Microbiol. Ecol..

[11] Winn M., Foulkes J.M., Perni S., Simmons M.J.H., Overton T.W., Goss R.J.M. (2012). Biofilms and their engineered counterparts: A new generation of immobilised biocatalysts. Catal. Sci. Technol..

[12] Perni S., Hackett L., Goss R.J., Simmons M.J., Overton T.W. (2013). Optimisation of engineered Escherichia coli biofilms for enzymatic biosynthesis of l-halotryptophans. AMB Express.

[13] Campoccia D., Montanaro L., Arciola C.R. (2013). A review of the biomaterials technologies for infection-resistant surfaces. Biomaterials.

[14] Sjollema J., Zaat S.A., Fontaine V., Ramstedt M., Luginbuehl R., Thevissen K., Li J., Van Der Mei H.C., Busscher H.J. (2018). In vitro methods for the evaluation of antimicrobial surface designs. Acta Biomater..

[15] Tambe S.M., Sampath L., Modak S.M. (2001). In vitro evaluation of the risk of developing bacterial resistance to antiseptics and antibiotics used in medical devices. J. Antimicrob. Chemother..

[16] Chung K.K., Schumacher J.F., Sampson E.M., Burne R.A., Antonelli P.J., Brennan A.B. (2007). Impact of engineered surface microtopography on biofilm formation of Staphylococcus aureus. Biointerphases.

[17] Wu S.Z., Zhang B.T., Liu Y., Suo X.K., Li H. (2018). Influence of surface topography on bacterial adhesion: A review. Biointerphases.

[18] Feng G., Cheng Y., Wang S.-Y., Borca-Tasciuc D.A., Worobo R.W., Moraru C.I. (2015). Bacterial attachment and biofilm formation on surfaces are reduced by small-diameter nanoscale pores: How small is small enough?. NPJ Biofilms Microbiomes.

[19] Rizzello L., Cingolani R., Pompa P.P. (2013). Nanotechnology tools for antibacterial materials. Nanomedicine.

[20] Scardino A.J., de Nys R. (2011). Mini review: Biomimetic models and bioinspired surfaces for fouling control. Biofouling.

[21] Hasan J., Jain S., Padmarajan R., Purighalla S., Sambandamurthy V.K., Chatterjee K. (2018). Multi-scale surface topography to minimize adherence and viability of nosocomial drug-resistant bacteria. Mater. Des..

[22] Elbourne A., Crawford R.J., Ivanova E.P. (2017). Nano-structured antimicrobial surfaces: From nature to synthetic analogues. J. Colloid Interface Sci..

[23] Lin N., Berton P., Moraes C., Rogers R.D., Tufenkji N. (2018). Nanodarts, nanoblades, and nanospikes: Mechano-bactericidal nanostructures and where to find them. Adv. Colloid Interface Sci..

[24] Modaresifar K., Azizian S., Ganjian M., Fratila-Apachitei L.E., Zadpoor A.A. (2019). Bactericidal effects of nanopatterns: A systematic review. Acta Biomater..

[25] Wu S.M., Zuber F., Maniura-Weber K., Brugger J., Ren Q. (2018). Nanostructured surface topographies have an effect on bactericidal activity. J. Nanobiotechnol..

[26] Valle J., Burgui S., Langheinrich D., Gil C., Solano C., Helbig R., Lasagni A., Lasa I., Toledo-Arana A., Toledo-Arana A. (2015). Evaluation of Surface Microtopography Engineered by Direct Laser Interference for Bacterial Anti-Biofouling. Macromol. Biosci..

[27] Günther D., Friedrichs J., Rößler F., Helbig R., Lasagni A., Werner C. (2016). The impact of structure dimensions on initial bacterial adhesion. Biomater. Sci..

[28] Rosenkranz A., Hans M., Gachot C., Thome A., Bonk S., Mücklich F. (2016). Direct Laser Interference Patterning: Tailoring of Contact Area for Frictional and Antibacterial Properties. Lubricants.

[29] Gillett A., Waugh D., Lawrence J., Swainson M., Dixon R. (2016). Laser surface modification for the prevention of biofouling by infection causing Escherichia Coli. J. Laser Appl..

[30] Bonse J., Krüger J., Höhm S., Rosenfeld A. (2012). Femtosecond laser-induced periodic surface structures. J. Laser Appl..

[31] Vorobyev A.Y., Guo C.L. (2013). Direct femtosecond laser surface nano/microstructuring and its applications. Laser Photonics Rev..

[32] Heitz J., Arenholz E., Sauerbrey R., Phillips H.M. (1994). Femtosecond excimer-laser-induced structure formation on polymers. Appl. Phys. A.

[33] Mezera M., van Drongelen M., Römer G.R.B.E. (2018). Laser-Induced Periodic Surface Structures (LIPSS) on Polymers Processed with Picosecond Laser Pulses. J. Laser Micro Nano..

[34] Rebollar E., Ezquerra T.A., Castillejo M., Mihailescu I.N., Caricato A.P. (2018). Laser Nanostructuring of Polymers. Pulsed Laser Ablation: Advances and Applications in Nanoparticles and Nanostructuring Thin Films.

[35] Danso D., Chow J., Streit W.R. (2019). Plastics: Microbial Degradation, Environmental and Biotechnological Perspectives. Appl. Environ. Microbiol..

[36] Pruitt L., Furmanski J. (2009). Polymeric biomaterials for load-bearing medical devices. JOM.

[37] Maitz M.F. (2015). Applications of synthetic polymers in clinical medicine. Biosurf. Biotribol..

[38] Rossetti F.F., Siegmann K., Köser J., Wegner I., Keskin I., Schlotterbeck G., Winkler M. (2017). Antimicrobial Polyethylene through Melt Compounding with Quaternary Ammonium Salts. Int. J. Polym. Sci..

[39] Zhang W., Zhang Y., Ji J., Yan Q., Huang A., Chu P.K. (2007). Antimicrobial polyethylene with controlled copper release. J. Biomed. Mater. Res. Part A.

[40] Seyfriedsberger G., Rametsteiner K., Kern W. (2006). Polyethylene compounds with antimicrobial surface properties. Eur. Polym. J..

[41] Erdmann M., Böhning M., Niebergall U. (2019). Physical and chemical effects of biodiesel storage on high-density polyethylene: Evidence of co-oxidation. Polym. Degrad. Stab..

[42] Rédei G.P. (2008). Encyclopedia of Genetics, Genomics, Proteomics and Informatics.

[43] Schindelin J., Arganda-Carreras I., Frise E., Kaynig V., Longair M., Pietzsch T., Preibisch S., Rueden C., Saalfeld S., Schmid B. (2012). Fiji: An open-source platform for biological-image analysis. Nat. Methods.

[44] Zhang X.X., Wang L., Levanen E. (2013). Superhydrophobic surfaces for the reduction of bacterial adhesion. RSC Adv..

[45] Hasan J., Raj S., Yadav L., Chatterjee K. (2015). Engineering a nanostructured “super surface” with superhydrophobic and superkilling properties. RSC Adv..

[46] Yuan Y., Hays M.P., Hardwidge P.R., Kim J. (2017). Surface characteristics influencing bacterial adhesion to polymeric substrates. RSC Adv..

[47] Falde E.J., Yohe S.T., Colson Y.L., Grinstaff M.W. (2016). Superhydrophobic materials for biomedical applications. Biomaterials.

[48] Hwang G.B., Page K., Patir A., Nair S.P., Allan E., Parkin I.P. (2018). The Anti-Biofouling Properties of Superhydrophobic Surfaces are Short-Lived. ACS Nano.

[49] Lamb R., Zhang H., Lewis J. (2005). Engineering nanoscale roughness on hydrophobic surface—Preliminary assessment of fouling behaviour. Sci. Technol. Adv. Mater..

[50] Lutey A.H.A., Gemini L., Romoli L., Lazzini G., Fuso F., Faucon M., Kling R. (2018). Towards Laser-Textured Antibacterial Surfaces. Sci. Rep..

[51] Hsu L.C., Fang J., Borca-Tasciuc D.A., Worobo R.W., Moraru C.I. (2013). Effect of Micro- and Nanoscale Topography on the Adhesion of Bacterial Cells to Solid Surfaces. Appl. Environ. Microbiol..

[52] Fadeeva E., Truong V.K., Stiesch M., Chichkov B.N., Crawford R.J., Wang J., Ivanova E.P. (2011). Bacterial Retention on Superhydrophobic Titanium Surfaces Fabricated by Femtosecond Laser Ablation. Langmuir.

[53] Epperlein N., Menzel F., Schwibbert K., Koter R., Bonse J., Sameith J., Krüger J., Toepel J. (2017). Influence of femtosecond laser produced nanostructures on biofilm growth on steel. Appl. Surf. Sci..

[54] Hou S., Gu H., Smith C., Ren D. (2011). Microtopographic Patterns Affect Escherichia coli Biofilm Formation on Poly(dimethylsiloxane) Surfaces. Langmuir.

[55] Gu H., Chen A., Song X., Brasch M.E., Henderson J.H., Ren D. (2016). How Escherichia coli lands and forms cell clusters on a surface: a new role of surface topography. Sci. Rep..

[56] Epstein A.K., Hochbaum A.I., Kim P., Aizenberg J. (2011). Control of bacterial biofilm growth on surfaces by nanostructural mechanics and geometry. Nanotechnology.

[57] Friedlander R.S., Vlamakis H., Kim P., Khan M., Kolter R., Aizenberg J. (2013). Bacterial flagella explore microscale hummocks and hollows to increase adhesion. Proc. Natl. Acad. Sci. USA.

